# Fecal Excretion of *Mycobacterium leprae*, Burkina Faso

**DOI:** 10.3201/eid2706.200748

**Published:** 2021-06

**Authors:** Anselme Millogo, Ahmed Loukil, Coralie L’Ollivier, Diakourga Arthur Djibougou, Sylvain Godreuil, Michel Drancourt

**Affiliations:** Centre Hospitalier Universitaire Souro Sanou, Bobo-Dioulasso, Burkina Faso (A. Millogo);; Aix-Marseille-Université, Institut de recherche pour le développement, Institut Hospitalo-Universitaire Méditerranée Infection, Marseille, France (A. Millogo, A. Loukil, M. Drancourt);; Université de Montpellier, Institut de Recherche pour le développement, Montpellier, France (A. Millogo, S. Godreuil);; Aix-Marseille-Université, IRD, Assistance Publique-Hopitaux de Marseille, Service de Santé des Arméés, Marseille (C. L’Ollivier);; Institut de Recherche en sciences de la Santé, Bobo-Dioulasso (D.A. Djibougou);; Centre MURAZ, Bobo-Dioulasso (D.A. Djibougou)

**Keywords:** DDD, FISH, *Mycobacterium*, *Mycobacterium leprae*, bacteria, bacterial infections, leprosy, fecal excretion, stool specimens, Burkina Faso, tuberculosis and other mycobacteria

## Abstract

*Mycobacterium leprae* was detected by optical microscopy, fluorescent in situ hybridization, and molecular detection in feces collected for the diagnosis of *Entamoeba coli* enteritis in a leprosy patient in Burkina Faso. This observation raises questions about the role of fecal excretion of *M. leprae* in the natural history and diagnosis of leprosy.

Leprosy caused by *Mycobacterium leprae* remains endemic in Burkina Faso, a West Africa country with a level of disability 2 of 31.2% among new patient cases (*1*). Laboratory diagnosis of leprosy is determined by observation of acid-fast bacilli after microscopic examination of a Ziehl-Neelsen–stained nasal smears and cutaneous lesions (*1*). Recently, fluorescence in situ hybridization (FISH) was introduced as a complementary approach to increase the specificity of microscopic observations (*1*,*2*). We report on the specific microscopic detection of *M. leprae* in the stool specimen of a patient in Burkina Faso. 

A 20-year-old man originating from the village of Bama in Burkina Faso sought care at the dermatology department at the Centre Hospitalier Universitaire Souro Sanou (Bobo-Dioulasso, Burkina Faso) for multiple infiltrated papules and nodules on his face and ear pavilions. These symptoms were accompanied by rhinitis and nosebleeds, which had been evolving for >2 months. Clinical examination further showed nasal enlargement (papulonodular), ulcerative-crusted lesions on the limbs, ulnar nerve hypertrophy, and a sausage-like appearance of the fingers, all of which suggested a lepromatous form of leprosy. A nasal smear and skin biopsy were performed on an infiltrative lesion (right arm), and 3 swab specimens were collected from a skin wound (left forearm), crusted lesions (elbow of right arm), and ulcerative papules (left arm). All samples were microscopically examined after Ziehl-Neelsen staining and revealed acid-fast bacilli in all 5 samples. Acid-fast bacilli were further identified as *M. leprae* by partial PCR amplification sequencing of the *rpoB* gene using a validated protocol (*1*). 

The patient also had abdominal pain, and stool samples were collected to check for parasites. Microscopic examination (at 400× magnification) of fresh stool specimens mixed with Lugol’s solution revealed cysts containing >6 nuclei, suggesting cysts of *Entamoeba coli*. Microscopic examination of the stool specimens filtrate after Ziehl-Neelsen staining (at 60× magnification) revealed 2 acid-fast bacilli per 300 microscopic fields (Figure). 

**Figure:**
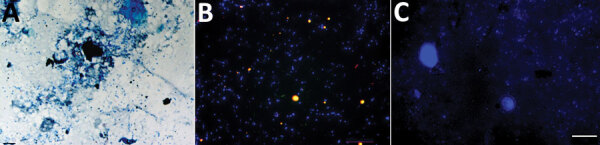
Optical microscopy observation of *Mycobacterium leprae* in the stool specimens of a leprosy patient in Burkina Faso. A) Ziehl-Neelsen staining; B) fluorescence in situ hybridization. No mycobacteria were observed inside the *Entamoeba coli* cysts (C). Scale bars represent 10 (A), 20 (B), and 20 (C) microns.

Identification of the pathogens was confirmed by a PCR-based method and FISH for *M. leprae* (Appendix). Because *M. leprae* has been identified as an intra-amoebal pathogen (*3*), we tested the intracystic location of *M. leprae* by FISH in clarified stool specimens using sucrose-density gradients. In brief, the cyst wall was permeabilized by incubating stool specimen in 1 mL of cellulase (Sigma Aldrich, https://www.sigmaaldrich.com) for 48 hours at 45°C (*4*). After cellulase activity was stopped by washing with physiologic water and 5 minutes of centrifugation at 3,000 g, the pellet was incorporated into 4’,6-diamidino-2-phenylindole-FISH staining. Observation of 8 *Escherichia coli* cysts disclosed nuclei stained with 4’,6-diamidino-2-phenylindole and an absence of any detectable *M. leprae* by FISH (Figure). Dynamic, dormant, and dead staining to identify the viability of mycobacteria (*5*) revealed dead mycobacteria in the skin biopsy, the 3 cutaneous swab specimens, and stool specimens, whereas 8 bacilli out of a total of 22 observed in a series of 6 microscopic fields in the nasal smear were dynamic (Appendix Figure).

Previous reports relied only on Ziehl-Neelsen staining to assess the presence of acid-fast bacilli in stool specimens collected from patients in whom leprosy was diagnosed, without any further formal identification (*6*,*7*). In the patient we report, stoolborne acid-fast bacilli were identified as *M. leprae* by 2 independent methods in the presence of negative controls. These *M. leprae* organisms were possibly swallowed by the patient along with blood or upper respiratory secretions during leprosy rhinitis and epistaxis (*7*). This observation correlates with a study in armadillos, an *M. leprae* host in some leprosy-endemic regions, in which experimental infection results in the extensive involvement of the intestine and the presence of *M. leprae* in stools (*8*). In the stool specimens of the patient described in this study, only dead *M. leprae* cultures were observed using dynamic, dormant, dead staining, whereas dynamic mycobacteria were detected in the nasal smear (*9*). 

On the basis of this research, further studies are required to confirm the prevalence of fecal excretion of *M. leprae* in various leprosy populations. Because stools are a noninvasive specimen, they could be collected for the positive diagnosis of leprosy using appropriate laboratory methods, as reported for the positive diagnosis of pulmonary tuberculosis (*10*).This diagnostic approach is easy to implement, including in children, in contrast to the current biopsy procedure, which requires a qualified staff and postsurgical management.

Appendix Additional information about fecal excretion of *Mycobacterium leprae*, Burkina Faso.
