# Supplementary material for: Fecal Excretion of Mycobacterium leprae, Burkina Faso
Source: Emerg Infect Dis. 2021 Jun;27(6):1758–60. doi: 10.3201/eid2706.200748 (PMC8153878; doi:10.3201/eid2706.200748)
Supplement: Appendix — Additional information about fecal excretion of Mycobacterium leprae, Burkina Faso. [file 20-0748-Techapp-s1.pdf]

Article DOI: https://doi.org/10.3201/eid2706.200748

Fecal Excretion of *Mycobacterium leprae*, Burkina Faso

Appendix

DNA was extracted from stool by combining chemical lysis, glass powder, heating at 56°C for 2.5 hours, sonication, and EZ1 automatic elution (QIAGEN, https://www.qiagen.com). *Escherichia coli* 18S rRNA partial PCR was performed using AmpliTaq GoldTM 360 Master Mix (Applied Biosystems, https://www.thermofisher.com) and incorporating forward primer: 5′-CGAGGAATAAGGGTTCGACA3′ and reverse primer: 5′-ATCGCTTTCTCACGATGGTT-3′ picked using Primer3Plus software (https://primer3plus.com/cgi-bin/dev/primer3plus.cgi) based on the reference *E. coli* sequence (GenBank accession no. MH133210.1) following the following thermal cycle: initial denaturation at 95°C for 15 minutes, 35 cycles of 30 seconds at 95°C, 30 seconds at 64°C and 1 minute at 72°C. Final extension at 72°C performed for 5 minutes. Molecular identification by real-time glbO PCR of *M. leprae* incorporated 6-FAM CGCGAGCCCGTCAGAATCTCCG6TAMRA, Mycoblep_1203 F: 5′-GGAATTTCGTCACAATTCCACAA-3′, Mycoblep_1203 R: 5′-TCGTCTCGTATCCGCCAATC-3′ in the presence of negative controls.

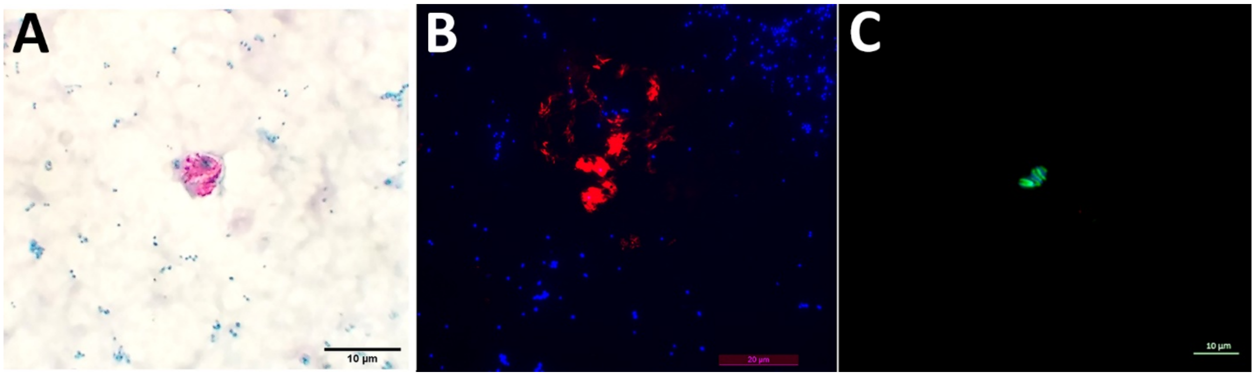

Appendix Figure. Optical microscopy observation of *Mycobacterium leprae* in the nasal swab of a Burkinabe patient after Ziehl-Neelsen staining (A), FISH staining (B), and DDD staining (C).
